# Isospecific Polymerization of 1‑Phenyl-1,3-butadiene and Its Copolymerization with Terpene-Derived Monomers

**DOI:** 10.1021/acs.macromol.5c00849

**Published:** 2025-07-01

**Authors:** Ilaria Grimaldi, Raffaele Marzocchi, Sara Esposito, Antonio Buonerba, Finizia Auriemma, Giuseppe Femina, Carmine Capacchione

**Affiliations:** † Dipartimento di Chimica e Biologia “Adolfo Zambelli”, 19028 Università degli Studi di Salerno, Via Giovanni Paolo II Fisciano, Salerno 84084, Italy; ‡ Dipartimento di Scienze Chimiche, 9307 Università di Napoli Federico II, Complesso Monte S. Angelo Via Cintia, Napoli 80126, Italy

## Abstract

The transition from fossil-based materials to biobased alternatives has become a critical research focus, particularly in the polymer sector, due to environmental concerns such as rising CO_2_ levels and microplastic pollution. This work explores the stereospecific polymerization of 1-phenyl-1,3-butadiene (1PB), a bioderived monomer from cinnamaldehyde, using a titanium [OSSO]-type catalyst activated by MAO. The polymerization exhibited high 3,4-regioselectivity and isotacticity (*mmmm* > 99%) with a maximum yield of 65% at 80 °C. Post-polymerization hydrogenation reduced the glass transition temperature (*T*_g_) from ≈80 °C to ≈17 °C, highlighting the impact of double bond removal on polymer flexibility. Additionally, copolymerizations of 1PB with natural terpenes β-ocimene (O) and *S*-4-isopropenyl-1-vinyl-1-cyclohexene (IVC) were conducted, yielding multiblock copolymers PPBO and PPBI, respectively, with tunable thermal properties. These copolymers showed partial cross-linking reactions and consequent presence of two glass transition temperatures (*T*_g_). For PPBO copolymers, the low *T*_g_ values tended to significantly decrease as the terpene content increased, whereas for the PPBI copolymers, the low *T*_g_ values showed minimal changes due to the similar *T*_g_ of their homopolymers. These findings demonstrate the potential of renewable monomers for producing sustainable polymers.

## Graphical abstract

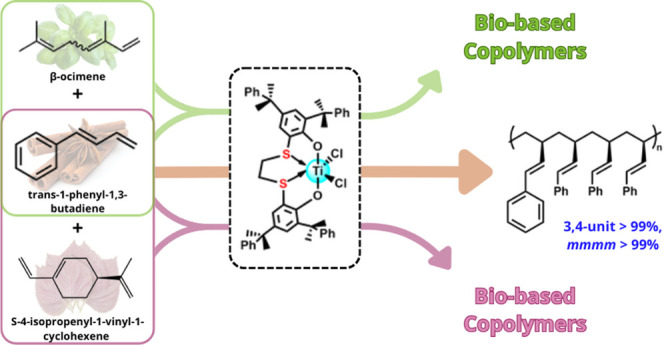

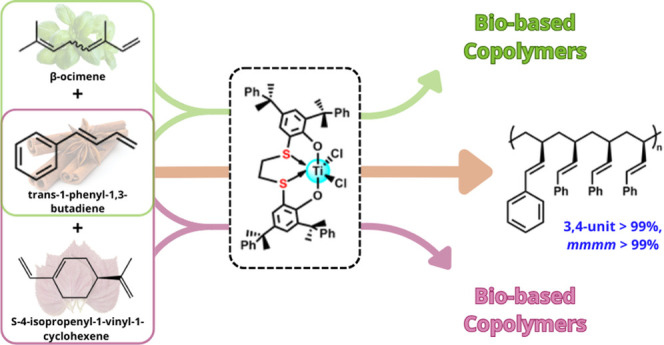

## 1 Introduction

The shift from a chemistry based on non-renewable resources to biobased alternatives has become an active task of research in the last years driven by the awareness of the negative effects of increasing CO_2_ concentration in the Earth’s atmosphere.^1–5^ In the case of polymeric materials, the search for new biopolymers is even more compelling than other sectors of the chemical industry because of the additional problem due to the widespread presence of microplastics in the environment that can have severe consequences on the ecosystems and, through the food chain, on human health.^6–8^ This situation has sparked the search for alternatives to synthetic polymers based on monomers coming from fossil resources, such as polyethylene, polypropylene, and synthetic rubbers.^9^ Indeed, the two most important synthetic rubbers are by far *cis*-1,4-polybutadiene and styrene–butadiene copolymers both based on monomers coming from petroleum, and in spite of the fact that natural rubber is produced from biomass, the importance of synthetic rubbers has grown during the decades due to the possibility of better tailoring the properties of the final material.^10–12^ In particular, the tool that opened the way to precise control over the polymer microstructure in the polymerization of conjugated dienes is, analogous to the α-olefin, the coordination polymerization promoted by transition metal complexes.^13,14^ Notably, by judicious choice of the catalytic system, it is possible for a given monomer to obtain different polymers with a high degree of stereocontrol, resulting in polymeric materials with distinct physical properties. Among the dienes originating from natural sources, 1-phenyl,3-butadiene (1PB) is particularly interesting, possessing not only two conjugated double bonds like butadiene but also an aromatic ring like styrene. 1PB can be conveniently derived by a Wittig reaction from cinnamaldehyde, contained in the essential oil of many plants such as or .^15^ Notwithstanding the interest in obtaining stereoregular polymers from this monomer, its polymerization in many cases results in sluggish reactivity with a low degree of stereocontrol.^15–19^ Only recently, Cui and coworkers reported the synthesis of syndiotactic-3,4-poly­(1PB) in the presence of rare-earth metal catalysts with high activity.^15^ Herein we report on the coordination polymerization of 1PB with high degree of 3,4-regioslectivity and isospecifity in the presence of homogeneous titanium [OSSO]-type complexes.^20,21^ In order to explore the possibility of obtaining copolymers completely based on monomers coming from renewable resources, we also report on the copolymerization of 1PB with various monomers coming from natural terpenes, such as ocimene (O) and *S*-4-isopropenyl-1-vinyl-1-cyclohexene (IVC), showing the potential to obtain polymeric materials with a wide range of properties completely based on monomers originating from biomass.

## 2 Experimental Section

### 2.1 Materials and Methods

Reagents and solvents were purchased from Sigma-Aldrich, Merck, or TCI Chemicals. Solvents were dried and distilled before use. All air- and/or water-sensitive compound manipulations were carried out using a glovebox or standard Schlenk techniques under an N_2_ atmosphere. Commercial-grade toluene (Sigma-Aldrich) was dried over calcium chloride, refluxed for 48 h under a nitrogen atmosphere over sodium, and distilled before use. Methylaluminoxane (MAO; 10 wt % solution in toluene; Sigma-Aldrich) was used as received. Dichloro­{1,4-dithiabutanediyl-2,2’-bis­[4,6-bis­(2-phenyl-2-propyl)­phenoxy]}­titanium complex **1** was synthesized according to the literature procedure.^22^

NMR spectra were recorded on a Bruker AM 300 spectrometer (300 MHz for ^1^H; 75 MHz for ^13^C), a Bruker AVANCE 400 spectrometer (400 MHz for ^1^H; 100 MHz for ^13^C), and a Bruker AVANCE III 600 spectrometer (600 MHz for ^1^H; 150 MHz for ^13^C). ^1^H and ^13^C chemical shifts are listed in parts per million (ppm), referenced to tetramethylsilane (TMS) by using the protio residual signal of the deuterated solvent. Spectra are reported as chemical shift (δ ppm), multiplicity, and integration. Multiplicity is abbreviated as follows: singlet (s), doublet (d), triplet (t), multiplet (m), broad (br), and overlapped (o). The number-average molecular weights (*M*_n_) and molecular weight distributions of polymers (dispersity, *Đ*) were evaluated by gel permeation chromatography (GPC) using an Agilent 1260 Infinity Series GPC chromatograph equipped with an RI, PLGPC 220 refractive index detector. All measurements were performed with THF as the eluent at a flow rate of 1.0 mL/min at 35 °C. Monodisperse poly­(styrene) polymers were used as calibration standards. Differential scanning calorimetry (DSC) analyses were carried out with a Mettler Toledo DSC-822 apparatus in a flowing N_2_ atmosphere at a rate of 10 °C/min. X-ray powder diffraction (XRD) profiles were collected in reflection mode using a multipurpose PANalytical Empyrean diffractometer with Cu Kα radiation (λ = 0.15418 nm). Melt-pressed films (thickness = 340 μm) were prepared by melting selected as polymerized samples at ≈150 °C under pressure lower than 5 bar, to avoid preferred orientations of the chains, and cooling to room temperature at a rate of about 20 °C/min by circulation of cold water in press plates. Mechanical tests were carried out at room temperature by stretching specimens cut from the melt-pressed films (gauge length = 10 mm, width 3 mm), using a universal testing machine (Instron 5566H1543), according to ASTM D 412-87. The reported stress–strain curves and the values of the mechanical parameters are averaged over at least five independent experiments.

### 2.2 Monomer Synthesis

*trans*-1-Phenyl-1,3-butadiene was synthesized according to the literature.^15^ Under a nitrogen atmosphere, *n*-butyllithium (40.0 mmol, 16 mL, 2.5 M in hexane) was added dropwise over 30 min to a suspension of methyltriphenylphosphonium bromide (14.3 g, 40.0 mmol) in anhydrous THF (250 mL) at 0 °C. The mixture was stirred at 0 °C for an additional 2 h. Subsequently, cinnamaldehyde (4.2 g, 32.0 mmol) was added dropwise, and the reaction was allowed to proceed at room temperature for 22 h. Once the reaction was complete, the reaction mixture was neutralized with saturated NH_4_Cl, extracted with hexane, dried over Na_2_SO_4_, filtered, and concentrated under reduced pressure. The pure product was obtained in 72% yield via column chromatography on silica gel using hexane as the eluent. ^1^H NMR (300 MHz, CDCl_3_): δ 7.34 (d, *J* = 8.0 Hz, 1H), 7.25 (t, *J* = 7.5 Hz, 1H), 7.19–7.10 (m, 1H), 6.73 (dd, *J* = 15.6, 10.5 Hz, 1H), 6.55–6.34 (m, 1H), 5.27 (d, *J* = 16.8 Hz, 1H), 5.11 (d, *J* = 9.7 Hz, 1H).

### 2.3 Polymerization of 1-Phenyl-1,3-butadiene (Runs 1–6, Table 1)

Complex **1** (10 μmol) was added to a 10 mL Schlenk tube equipped with a magnetic stirrer and dissolved in 3 mL of dry toluene. MAO was added, and the solution was left stirring for 30 min to preactivate the metal complex. Then, 1PB (2.3 mmol, 0.3 g) was added, and the system was placed in an oil bath thermostated to the desired temperature (25, 40, or 80 °C) and stirred for the required time. The polymers were coagulated in an excess of acidified methanol containing 2,6-di*tert*-butyl-4-methylphenol (BHT) as an antioxidant, washed several times with methanol, recovered by filtration, and dried in a vacuum oven overnight.

**Table 1:** Homopolymerization of 1PB in the Presence of Catalyst **1** Activated with MAO

Run^a^	Sample	*T* (°C)	Time (h)	Yield (%)	*M*_n_ (kg/mol)^b^	*Đ* ^*b*^	*T*_g_ (°C)^c^
1	PPB1	25	15	12	28.8	1.5	85.0
2	PPB2	40	15	35	37.8	1.9	94.8
3	PPB3	80	15	58	31.0	2.1	83.7
4	PPB4	80	5	54	22.4	2.1	80.4
5	PPB5	80	24	65	21.0	2.3	84.0
6	PPB6	80	48	63	22.5	2.1	88.9

a Reaction conditions: Catalyst **1** (1.0 × 10^–5^ mol), [Al]/[Ti] = 500, 1PB (2.3 mmol, 0.3 g), toluene (5 mL).

b Determined by GPC.

c Determined by DSC in the 2nd heating scan.

### 2.4 Hydrogenation of Poly­(1PB)

The hydrogenation of poly­(1PB) was carried out in accordance with a literature procedure.^15^ A 100 mg sample of 3,4-isotactic poly­(1PB) (run 3, Table 1) was dissolved in 5 mL of *o*-xylene. An excess of *p*-toluenesulfonylhydrazine was added, and the mixture was stirred under reflux for 12 h. The fully reduced polymer was subsequently precipitated in methanol, filtered, and dried under vacuum overnight.

### 2.5 Copolymerization of 1PB and β-Ocimene/IVC (Runs 1–6, Table 2)

Complex **1** (5 μmol) was added into a 10 mL Schlenk tube equipped with a magnetic stirrer and dissolved in 4 mL of dry toluene. MAO was added, and the solution was left stirring for 30 min to preactivate the metal complex. Then, both comonomers (1PB and ocimene/IVC) were dissolved in 1 mL of dry toluene and added, and the system was placed in a thermostated oil bath at 80 °C and stirred for 24 h. The polymers were coagulated in an excess of acidified methanol containing 2,6-di*tert*-butyl-4-methylphenol (BHT), washed several times with methanol, recovered by filtration, and dried in a vacuum oven overnight.

**Table 2:** Copolymerization of 1PB and Terpenes in the Presence of Catalysts **1**/MAO^a^ ^c^ ^d^

						Polymer composition (**%**)^c^		
Run^a^	Sample	Terpene/mol %	Yield (%)	*M*_n_ (kg/mol)^b^	*Đ* ^b^	Terp. (mol %)	1PB (mol %)	*T*_g_ (°C)^d^
1	PPBO1	O/50	56	40.4	2.3	46	54	61.8
2	PPBO2	O/70	45	29.6	2.0	64	36	43.7/133.4
3	PPBO3	O/80	56	37.8	2.1	81	19	–20.4
4	PPBI1	IVC/50	98	152.4	1.9	51	49	71.9/106.1
5	PPBI2	IVC/70	>99	149.4	2.5	68	32	72.5/114.5
6	PPBI3	IVC/80	96	162.3	2.4	79	21	70.4

a Reaction conditions: catalyst (5.0 × 10^–6^ mol), ([1PB] + [Terp])/[Ti] = 1000, [Al]/[Ti] = 500, toluene (5 mL), 80 °C, 24 h.

b Determined by GPC.

c Determined by ^1^H and ^13^C NMR spectroscopy.

d Determined by DSC in the 2nd heating scan.

## 3 Results and Discussion

The stereospecific polymerization of 1-phenyl-1,3-butadiene (1PB), promoted by a titanium complex with [OSSO]-type ligand featuring cumyl substituents on the aromatic rings (complex **1**), was investigated. When activated by methylaluminoxane (MAO), complex **1** demonstrated high regioselectivity toward 3,4-insertion and exhibited remarkable isoselectivity (Scheme 1A). The results are summarized in Table 1.

**Scheme 1:**
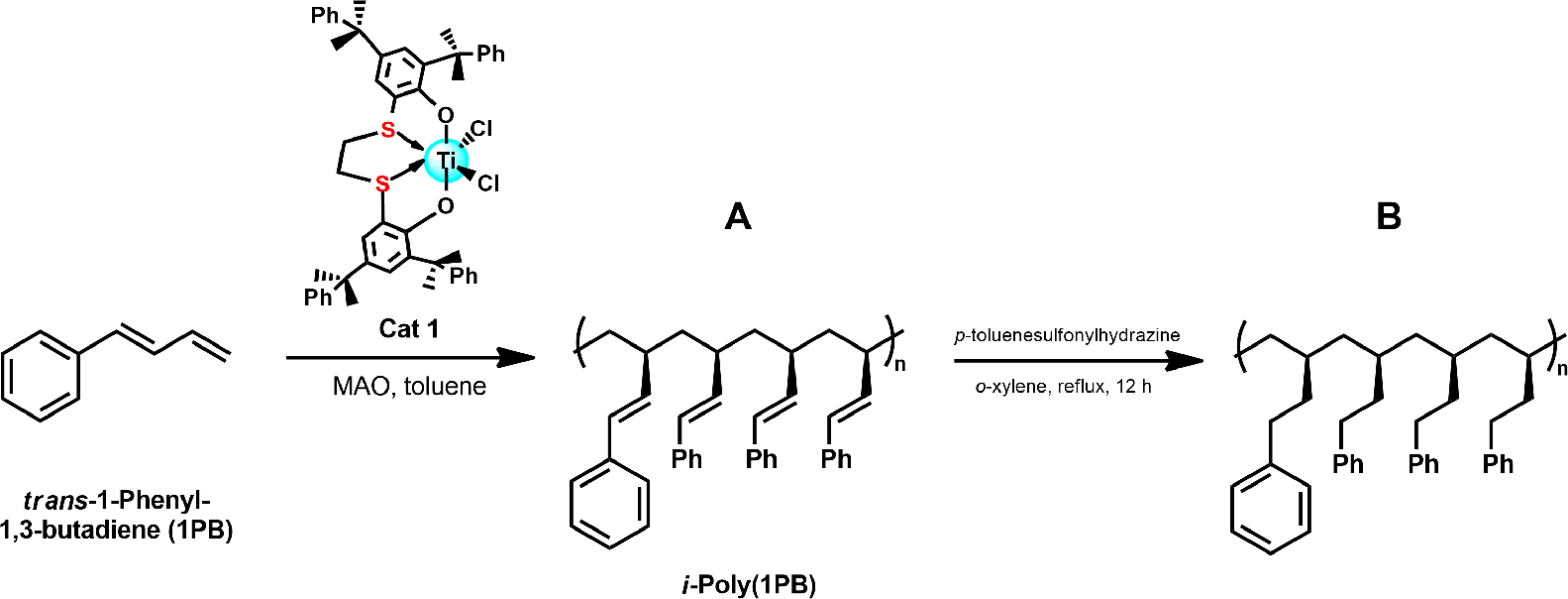
Isospecific Polymerization of 1PB Promoted by [OSSO]Titanium Complex **1** (A) and Subsequent Hydrogenation (B)

Catalyst **1** was employed in the synthesis of poly­(1-phenyl-1,3-butadiene) (poly­(1PB)) at various temperatures in toluene. The data (Table 1, runs 1–3) reveal that polymer yield increases with temperature, reaching a maximum of 58% at 80 °C after 15 h. In all cases, the obtained polymer exhibited high isotacticity and regioselectivity for 3,4-insertion (>99%), as confirmed by ^1^H and ^13^C NMR analyses (Figures 1 and S2). The ^13^C NMR spectrum shows a peak at 40.39 ppm, which is unambiguously assigned to the methylene carbon of the polymer backbone. The absence of other peaks, as reported for the syndiotactic polymer,^15^ indicates that the polymer is highly isotactic, with a pentad distribution of *mmmm* > 99%. At a fixed temperature of 80 °C, the reaction was monitored at different time intervals of 5, 24, and 48 h. The results indicate that the maximum yield (65%) was achieved after 24 h, without further variations. The so-reached quasi-plateau could be attributed to catalyst deactivation over time or the establishment of a dynamic equilibrium, where monomer consumption is balanced by chain termination or transfer processes.

**Figure 1:**
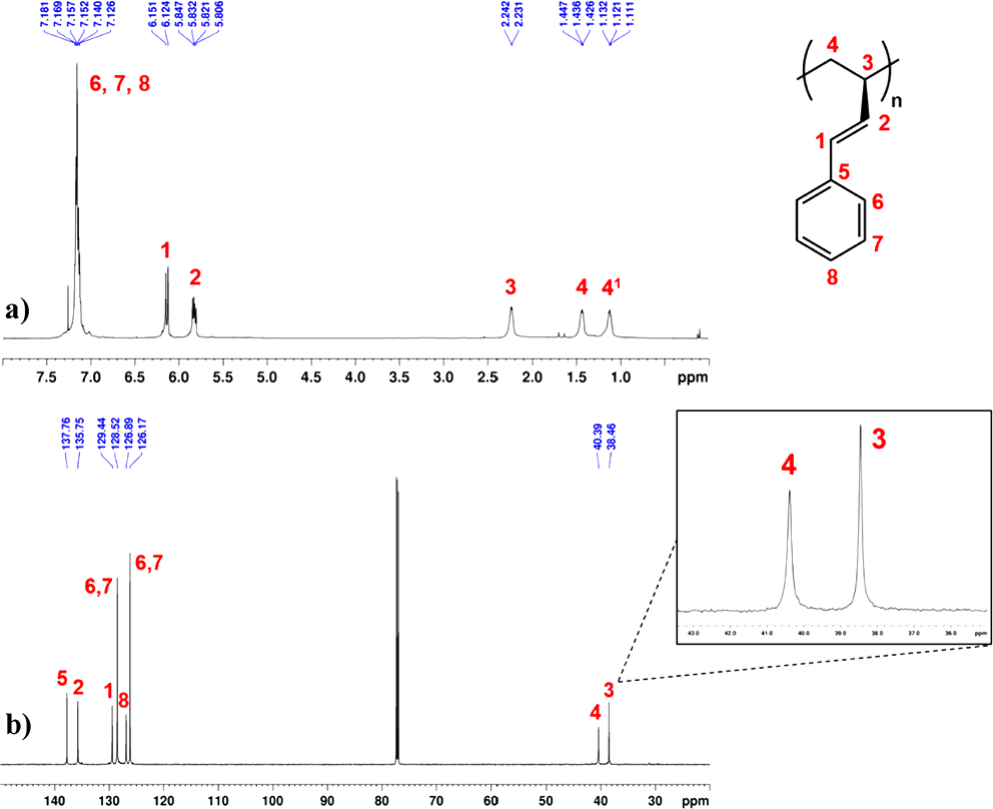
(a) ^1^H NMR (600 MHz, CDCl_3_, 298 K) and (b) ^13^C NMR (150 MHz, CDCl_3_, 298 K) of 3,4-isotactic poly­(1PB) from run **3**, Table 1.

The ability to control molecular weights aligns well with the characteristic behavior of [OSSO]-type complexes functioning as single-site catalysts in the polymerization of diene monomers.^23–27^ This is particularly evident from the dispersity (*Đ*) values obtained through gel permeation chromatography (GPC) analyses, which highlight the uniformity of the produced polymer chains (*Đ* = 19-2.5). The observed dispersity values are indicative of a well-regulated polymerization process, where each active site operates independently and uniformly.

The glass transition temperature (*T*_g_) values (*T_g_* = 85.0-94.8) are significantly lower compared to the *T*_g_ of the highly syndiotactic polymer (104 °C) produced using rare-earth-based catalysts.^15^ This difference can be attributed to the distinct stereoregularity of the polymer chains. In syndiotactic polymers, the alternating spatial arrangement of substituents along the polymer backbone enhances chain packing efficiency and intermolecular interactions, particularly π–π stacking between phenyl groups. This tighter packing and increased cohesion resulted in a higher *T*_g_. In contrast, isotactic polymers, despite their regular structure, may exhibit reduced chain packing efficiency due to steric hindrance caused by the uniform orientation of the phenyl substituents. This can lead to greater chain mobility and weaker intermolecular forces, ultimately contributing to a slightly lower *T*_g_ compared to their syndiotactic counterparts. A similar trend is observed in polystyrene, where the syndiotactic form exhibits a higher *T*_g_ than that of its isotactic counterpart. In syndiotactic polystyrene, the alternating phenyl groups enhance rigidity due to interchain interactions, while isotactic polystyrene exhibits greater chain mobility in the amorphous regions, contributing to a lower *T*_g_.^28,29^ This comparison illustrates how the stereoregularity of polymers significantly influences their thermal and mechanical properties.

As an example, the powder X-ray diffraction (XRD) profiles and the DSC thermograms of the as-synthesized PPB3 and PPB4 samples are shown in Figures 2 and S13 (curves a, b). It is apparent that the XRD profiles of the samples PPB3 and PPB4 were dominated by a main halo centered at 2θ ≈ 18°, preceded by a weak broad peak centered at 2θ ≈ 8°, corresponding to intra- and interchain correlation distances of ≈0.5 and 1 nm, respectively. The absence of relevant Bragg’s peaks indicates that the samples were essentially amorphous. Moreover, while the sample PPB3 showed two faint diffraction peaks at 2θ ≈ 24° and 35°, possibly due to the presence of a small crystalline fraction, for the sample PPB4 these peaks were not observed, probably because the crystals had a very small size.

**Figure 2:**
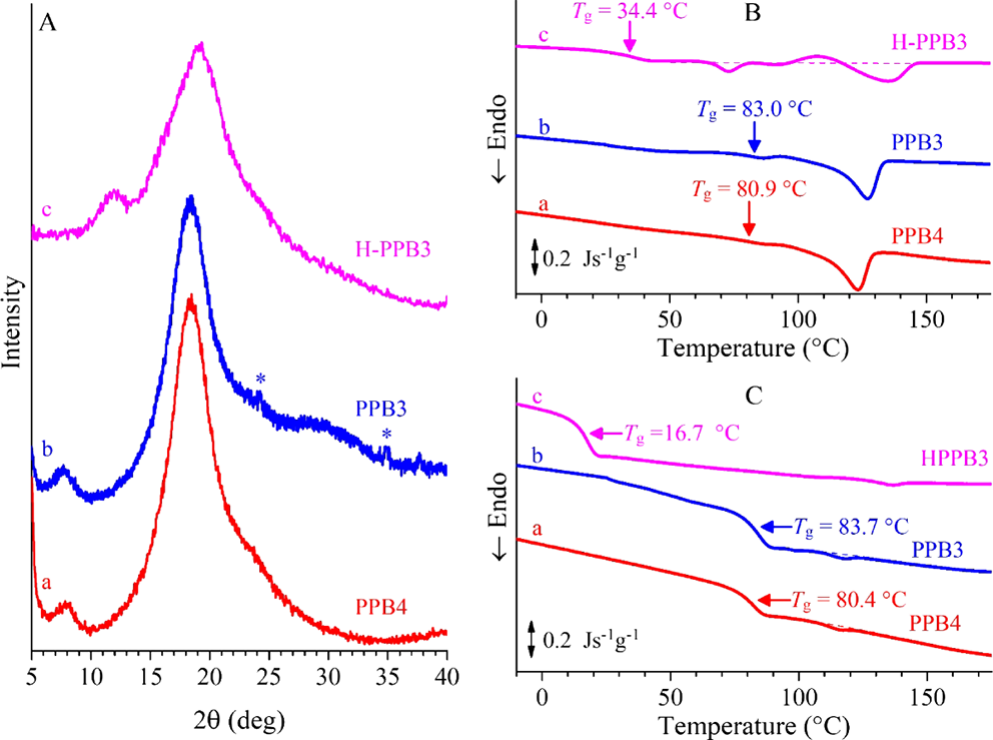
X-ray powder diffraction profiles of as-synthesized samples PPB4 (a), PPB3 (b) and of the sample PPB3 after hydrogenation (HPPB3, c) (A) and corresponding DSC curves recorded in the 1st (B) and 2nd heating (C). The star in A (curve b) marks weak peaks tracing back the presence of a small fraction of a crystalline phase.

The DSC curves of the samples PPB3 and PPB4 showed, in the first heating scan, a glass transition temperature at ≈80 °C and an endothermic peak around 125 °C (Figure 2B) with an area corresponding to ≈18 J/g (Table S1). The endotherm peak at ≈125 °C indicates the presence of a small crystalline fraction, even though the presence of these crystals could not be readily recognized through XRD analysis because they were of very small size (cryptocrystallinity).^30,31^ In the successive cooling scan, the DSC curves did not show any relevant exothermic peak but only the glass transition (curves a, b of Figure S13). However, the DSC curves recorded in the second heating scan showed, besides the glass transition at ≈80 °C (Table S1), a faint endothermic peak at ≈118 °C (Δ*H* ≈ 2 J/g), due to the melting of the cryptocrystals.

A sample of 3,4-isotactic (poly1PB) (run 3, PPB3, Table 1) with a *T*_g_ of approximately 84 °C was hydrogenated using *p*-toluenesulfonylhydrazine under reflux in *o*-xylene (Scheme 1B). Complete reduction of the double bonds was confirmed by ^1^H and ^13^C NMR spectra (see Figures S9 and S10). The resulting polymer HPPB3 (poly-4-phenyl-1-butene) retained its stereoregularity but exhibited a considerably lower *T*_g_ (approximately 17 °C), as observed by DSC analysis (curve c of Figure 2C and Table S1). The drastic decrease in *T*_g_ after the hydrogenation of the polymer double bonds could be attributed to the increased flexibility of the polymer backbone. Indeed, the damped rotational dynamics of the side chains, in which the −C=C– double bonds are conjugated to phenyl rings, induce a high glass transition temperature in the initial polymer species. The reduction of the double bonds to single bonds eliminates rotational restriction, enhancing chain mobility and thereby lowering the *T*_g_. Besides, the DSC curve recorded in the first heating scan (curve c of Figure 2B) showed a first endotherm at ≈76 °C (Δ*H* ≈ 3 J/g) due to the melting of a small crystalline fraction, followed by an exotherm at ≈109 °C (Δ*H* ≈ 3 J/g) due to recrystallization and a successive endotherm at ≈138 °C (Δ*H* ≈ 13 J/g), marking the complete melting of the crystals. The sample did not crystallize from the melt, as indicated by the DSC curve recorded in the cooling step (curve c in Figure S13) but tended to crystallize in part during the second heating scan, as indicated by the weak melting peak at ≈126 °C (Δ*H* ≈ 2 J/g) (curve c of Figure 2C and Table S1). The lack of any exothermic peak in the cooling and/or second heating DSC scans indicates that crystallization from the melt and/or the amorphous phase (cold crystallization) occurred over a broad range of temperatures above the *T*_g_, so that it could not be observed. It is worth noting that the presence of crystals in the as-synthesized HPPB3 samples was not detected in the corresponding XRD profile (curve c of Figure 2A). The hydrogenated sample, indeed, did not show Bragg’s peaks, probably because the size of the crystals was too low (cryptocrystallinity), but only two halos centered at 2θ ≈ 12° and 19° were present, corresponding to inter- and intrachain correlation distances of 0.7 and 0.5 nm, respectively. The increase of chain flexibility achieved by hydrogenated PPB, indeed, led to lower inter- and intrachain correlation distances than those achieved by the nonhydrogenated counterpart.

Isotactic poly-4-phenyl-1-butene was early synthesized from 4-phenyl-1-butene through TiCl_4_ in the presence of triethylaluminum as a catalyst system.^32^ The resulting polymer showed DSC curves similar to those of hydrogenated poly­(1PB) of Figure 2B (HPPB3, curve c), with a melting temperature of ≈158 °C.^32^ Specimens stretched up to 2–3 times their initial length and then annealed under vacuum for 1 week at 120 °C, suitable for X-ray fiber diffraction analysis, were then obtained. It was shown that the polymer crystallized with chains in a 3/1 helical conformation, packed in a monoclinic unit cell with orthorhombic parameters (*a* = 1.04 nm, *b* = 1.80 nm, *c* = 0.661 nm), according to the space group symmetry *Pa.* The results achieved in the present investigation demonstrate that isotactic poly­(4-phenyl-1-butene) may be obtained also by hydrogenation of isotactic PPB and highlight the impact of backbone structure on the thermal and mechanical behavior of polymers.

To further explore the synthesis of polymers derived from renewable sources, catalyst 1 was subsequently used in the copolymerization of 1PB with two natural monomers, β-ocimene (O), a terpene primarily found in plants like basil, and *S*-4-isopropenyl-1-vinyl-1-cyclohexene (IVC), which can be obtained from perillaldehyde, the main component of the perilla plant (Scheme 2). The development of such materials is of significant interest due to the growing demand for sustainable and environmentally friendly alternatives to traditional petrochemical-based polymers.

**Scheme 2:**
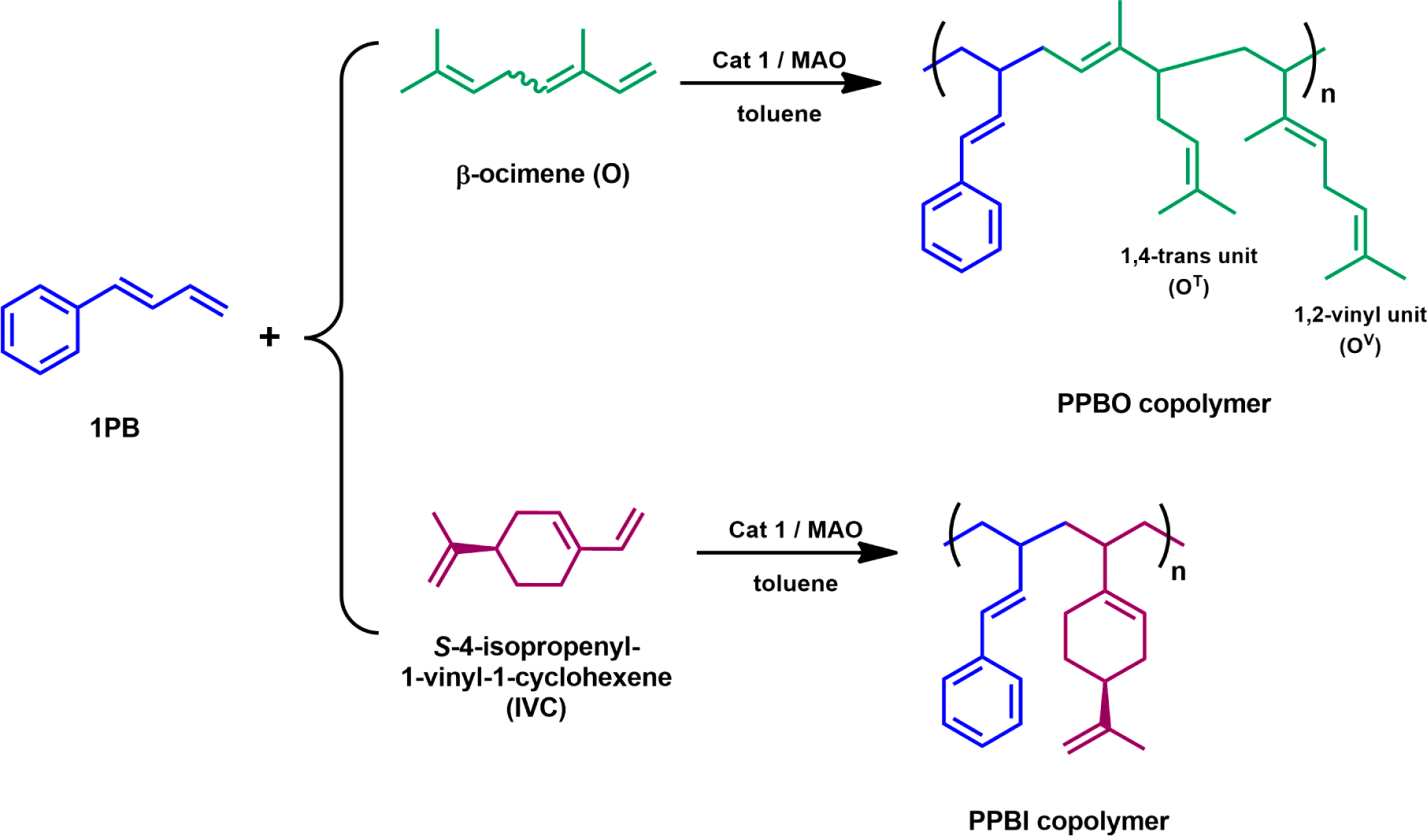
Copolymerization of 1PB and Terpenes Promoted by [OSSO]Titanium Complex (**1**) Activated with MAO

The catalytic system **1/MAO** has already demonstrated high efficiency in the homopolymerization of β-ocimene, standing out as one of the most effective systems for the synthesis of poly­(ocimene).^33^ Similarly, in the polymerization of IVC, the catalyst yielded a highly regioselective and isotactic polymer, showcasing its potential for producing precision-structured polymers from natural monomers.^34^

Several experiments were conducted by varying the composition of the two monomers in order to study the effect on the chemical, thermal, and mechanical properties of the resulting copolymers. O/1PB (PPBO) and IVC/1PB (PPBI) copolymers were hence obtained, as summarized in Table 2.

In the copolymerizations of 1PB with β-ocimene, the final polymer composition aligns with the molar ratio used between the two comonomers. The maximum conversion of 1PB is consistent with the conversion observed in homopolymerizations (approximately 60%), resulting in a polymer with lower yield in all three cases (runs 1–3, Table 2). The microstructure of all copolymers was thoroughly characterized by using a combination of analytical techniques, including NMR spectroscopy, gel permeation chromatography (GPC), and differential scanning calorimetry (DSC). The ^1^H and ^13^C NMR spectra (see Figures 3 and S3–S4) of the PPBO copolymers suggest a possible multiblock structure, where short homosequences of both comonomers alternate along the polymer chain. Indeed, both types of insertions of β-ocimene, 1,4-trans (O^T^) and 1,2-vinyl (O^V^), are observed, as expected.

**Figure 3:**
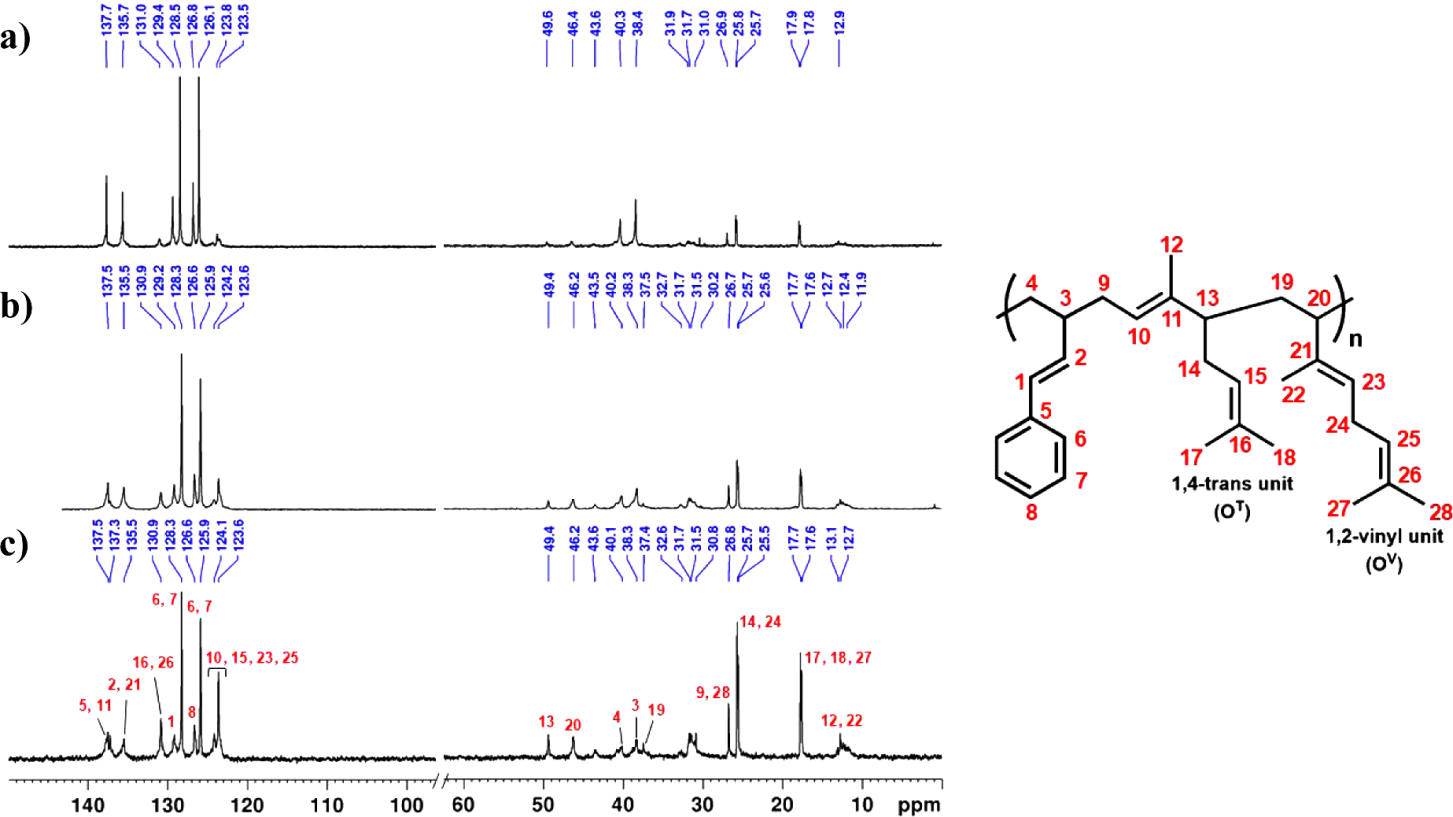
^13^C NMR spectra (100 MHz, CDCl_3_, 25 °C) of PPBO copolymers from (a) run **1** (PPBO1), (b) run **2** (PPBO2), and (c) run **3** (PPBO3) of Table 2.

In the copolymerizations with IVC, however, 1PB appears to be more reactive, as it is almost completely consumed (98–99%), achieving nearly quantitative yields. This increased reactivity could be explained by the potential influence of the comonomer in the coordination-insertion polymerization. Specifically, the presence of a comonomer might modify the coordination environment or the electronic structure of the catalytic sites, facilitating the insertion of 1PB into the growing polymer chain and thereby increasing its reactivity.^35^ The ^1^H and ^13^C NMR spectra suggest a multiblock structure also in this case (see Figures S6 and S7).

In all cases, the GPC analyses (see Figures S20 and S21) revealed monomodal profiles, and the *Đ* values confirmed the copolymeric nature of the samples, whereas the XRD profiles (Figure S14) indicate that all of the samples are amorphous. Additionally, the DSC curves recorded in the first heating scan (Figures S15) showed, at temperatures greater than ≈100 °C, the presence of spurious exothermic and/or endothermic peaks. These peaks are especially evident for the PPBO2 and PPBO3 samples (Figure S15A, A’). They are due to a portion of chain segments undergoing cross-link reactions and also, in part, to slight degradation. In particular, a slight degradation is indicated by the small weight loss occurring at temperatures lower than 200 °C in the thermogravimetric (TGA) traces of Figures S17 and S18. Moreover, the occurrence of cross-linking reactions was indicated by the presence of a glass transition in the second DSC heating traces (Figure S15C) of the samples PPBO2, PPBI, and PPBI2, with terpene content of 64, 51, and 68 mol %, at temperatures of ≈133, 106, and 144 °C, respectively, that is, at temperatures significantly greater than those of the homopolymers polyocimene,^33^ poly­(isopropenyl-1-vinyl-1-cyclohexene),^34^ and PPB. These samples showed a second glass transition at temperatures lower or similar to those of the corresponding homopolymers, indicating phase separation of cross-linked and non-cross-linked segments in different domains. The samples PPBO1, PPBO3, and PPBI3, with terpene unit content of 46, 81 and 79 mol %, respectively, instead showed a single glass transition temperature due to the good miscibility of terpene and 1PB sequences. Interestingly, for the uncross-linked fraction of the PPBO copolymers, the *T*_g_ values showed a marked decrease as the terpene content increased, suggesting a pronounced influence of the terpene incorporation on the thermal properties of the resulting polymers. In contrast, for the uncross-linked fraction of the PPBI copolymers, the effect on the glass transition temperature was less pronounced, as the two homopolymers exhibit similar *T*_g_ values.

Preliminary measurements of the mechanical properties of selected PPBO and PPBI samples, measured at room temperature, are shown in Figure S22, while the mechanical parameters are collected in Table S2. The samples showed values of deformation at break and Young’s modulus lower than 10% and greater than 130 MPa, respectively, indicating that they were rigid and fragile, in agreement with the high values of the glass transition temperature and the high intrinsic stiffness of the chains.

## 4 Conclusion

This work demonstrates the potential of renewable resources in the production of sustainable polymers through the stereospecific polymerization of 1-phenyl-1,3-butadiene (1PB) and its copolymerization with bioderived terpenes. The titanium [OSSO]-type catalyst (complex **1**), when activated by methylaluminoxane (MAO), proved highly effective in promoting 3,4-regioselective and isotactic polymerization of 1PB, achieving high stereocontrol (*mmmm* > 99%) and regioselectivity (>99%) under optimized conditions. Postpolymerization hydrogenation of isotactic poly­(1PB) led to a drastic reduction in the glass transition temperature (*T*_g_) from ≈80 to 17 °C, underscoring the critical role of backbone structure in determining thermal properties. The hydrogenation of the double bonds enhanced chain flexibility, transforming the polymer into a material with high potential to show elastomeric properties suitable for applications requiring elasticity and impact resistance at lower temperatures. The study also explored the copolymerization of 1PB with plant derived monomers β-ocimene and *S*-4-isopropenyl-1-vinyl-1-cyclohexene (IVC), yielding novel biobased copolymers with tunable thermal and mechanical properties. These copolymers tended to experience partial cross-linking at temperatures greater than 100 °C. For the non-cross-linked segments of PPBO copolymers, the incorporation of β-ocimene significantly influenced the *T*_g_, which decreased with increasing terpene content. In contrast, the non-cross-linked segments of PPBI copolymers showed less pronounced changes in *T*_g_, attributed to the similar glass transition temperatures of the two homopolymers. Interestingly, the copolymerization with IVC achieved nearly quantitative yields, as 1PB was almost completely consumed. The findings of this study highlight the versatility and efficiency of the [OSSO]-type titanium catalysts in the polymerization of bioderived monomers, opening the door to the design of sustainable polymers with properties tailored to specific applications. The ability to synthesize homopolymers and copolymers entirely from renewable resources addresses critical environmental challenges, including reliance on fossil fuels and microplastic pollution while offering innovative materials for advanced applications.

## Supplementary Material



## Notes

NMR characterization; DSC and XRD analyses; themogravimetric analyses; GPC analyses; mechanical properties (PDF)
