# Supplementary material for: Isospecific Polymerization of 1‑Phenyl-1,3-butadiene and Its Copolymerization with Terpene-Derived Monomers
Source: Macromolecules. 2025 Jul 1;58(15):8344–51. doi: 10.1021/acs.macromol.5c00849 (PMC12356061; doi:10.1021/acs.macromol.5c00849)
Supplement: Supplementary file 1 [file ma5c00849_si_001.pdf]

SUPPORTING INFORMATION

Isospecific Polymerization of 1-phenyl-1,3-butadiene and its Copolymerization with Terpene Derived Monomers

Ilaria Grimaldi, Raffaele Marzocchi[†], Sara Esposito, Antonio Buonerba, Finizia Auriemma[†,*], Giuseppe Femina[†] and Carmine Capacchione[*]

Dipartimento di Chimica e Biologia "Adolfo Zambelli", Università degli Studi di Salerno, Via Giovanni Paolo II, 84084 Fisciano, SA, Italy.

[†]Dipartimento di Scienze Chimiche Università di Napoli Federico II, Complesso Monte S. Angelo, Via Cintia, 80126, Napoli, Italy

*E-mail: ccapacchione@unisa.it; auriemma@unina.it

Table of content

1. **NMR characterization of the monomer** 2
2. **NMR characterization of the polymers and copolymers** 3
3. **Hydrogenation of poly(1PB)** 6
4. **DSC and XRD Analyses** 8
5. **Thermogravimetric Analyses** 10
6. **GPC Analyses** 12
7. **Mechanical properties** 13

References 14

1. NMR characterization of the monomer

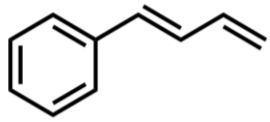

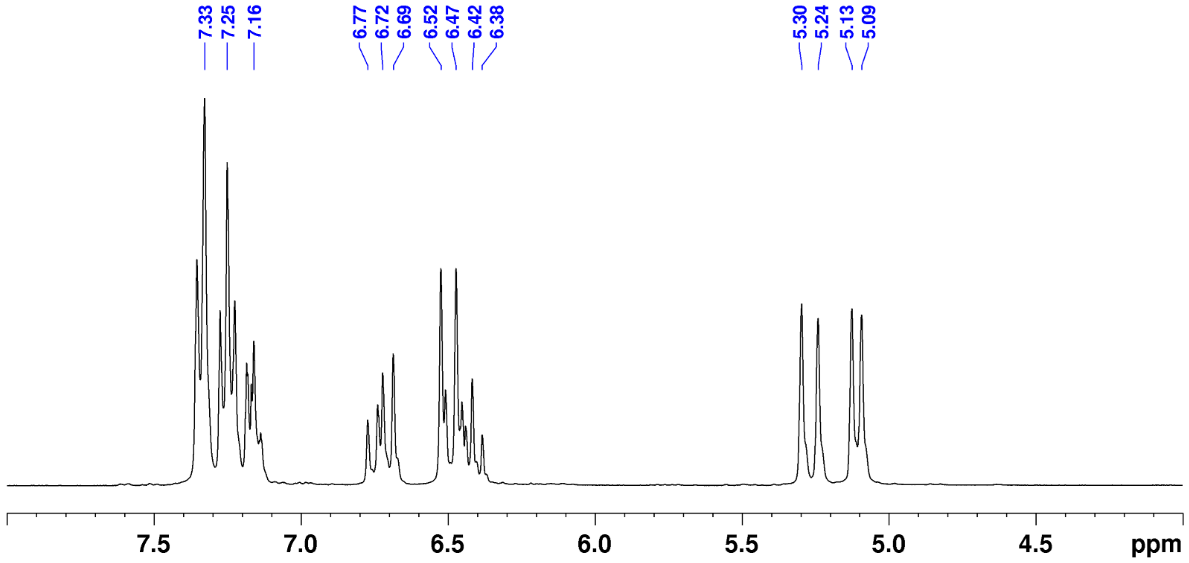

Figure S1. ^{1}H NMR (400 MHz, $CDCl_3$, 298 K) of *trans*-1-phenyl-1,3-butadiene (1PB).

2. NMR characterization of the polymers and copolymers

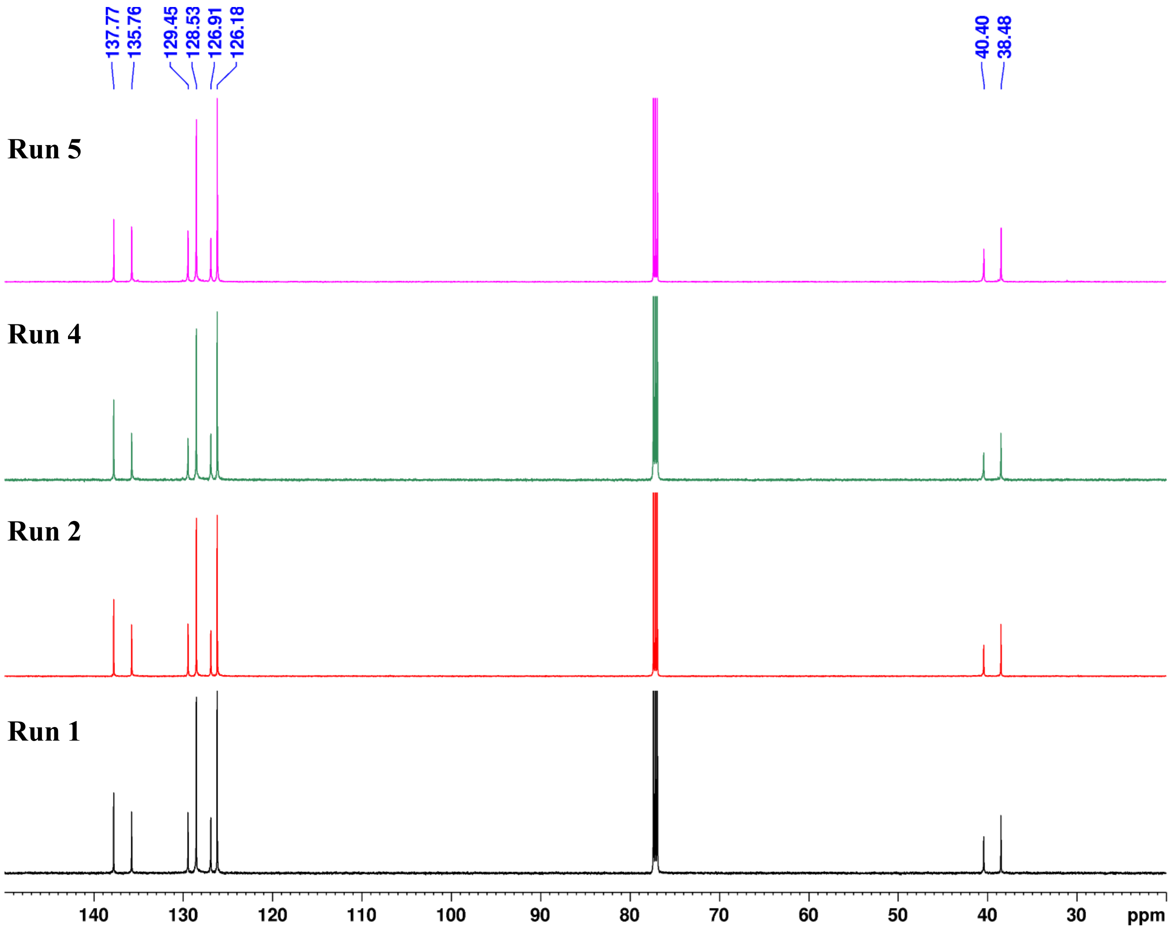

Figure S2. ^{13}C NMR (150 MHz, CDCl3, 298 K) of 3,4-isotactic poly(1PB) (Table 1, runs 1,2,4, and 5).

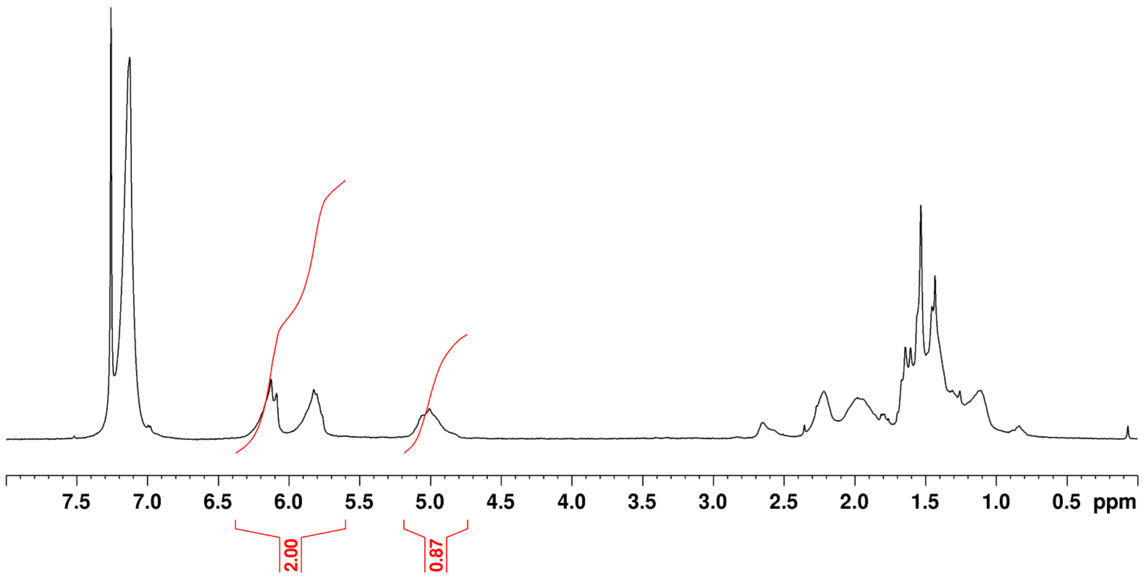

Figure S3. ^{1}H NMR (400 MHz, $CDCl_3$, 298 K) of PPBO copolymer from run **1**, Table 2.

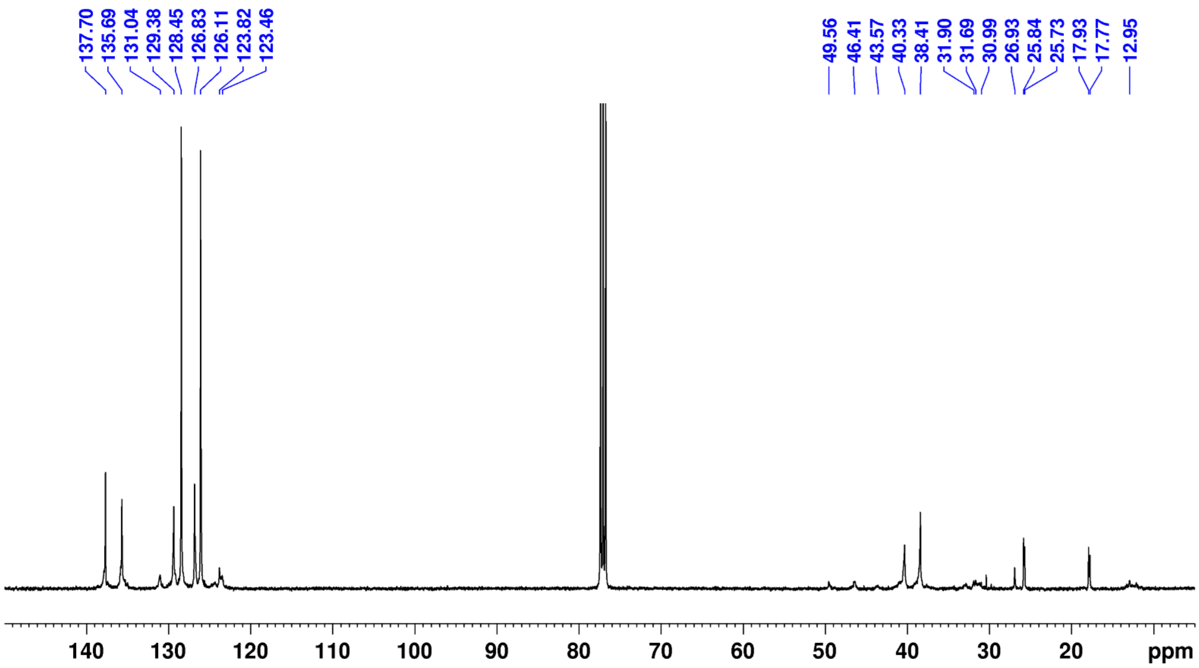

Figure S4. ^{13}C NMR (100 MHz, $CDCl_3$, 298 K) of PPBO copolymer from run **1**, Table 2.

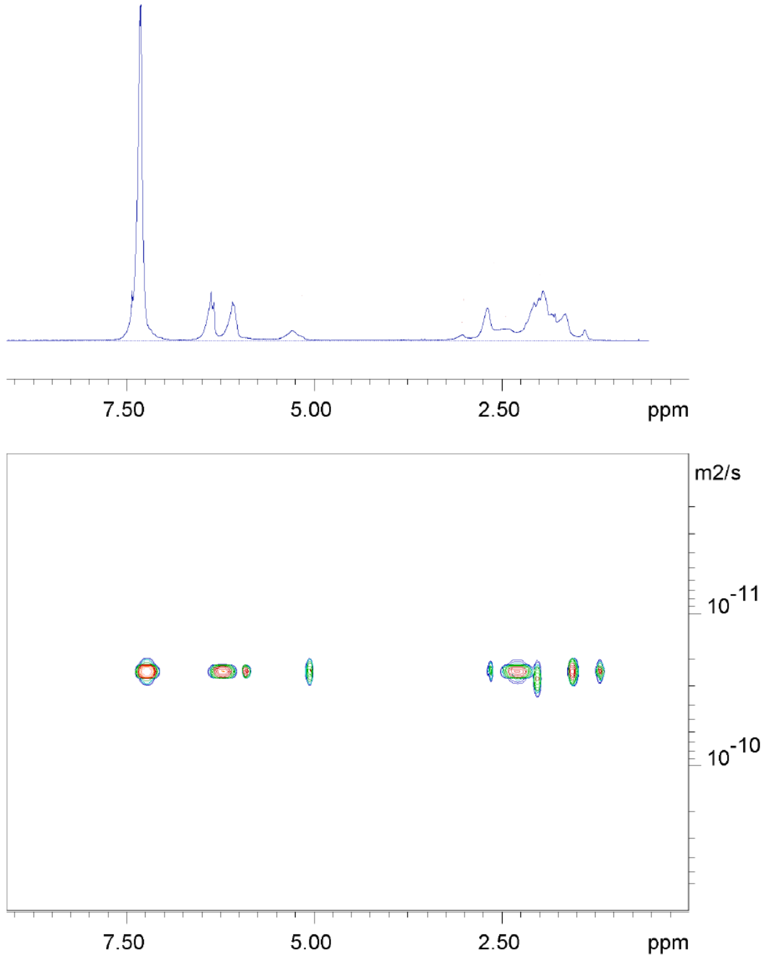

Figure S5. 2D DOSY NMR spectra (400 MHz, $CDCl_3$, 298 K) of PPBO copolymer from run **1**, Table 2.

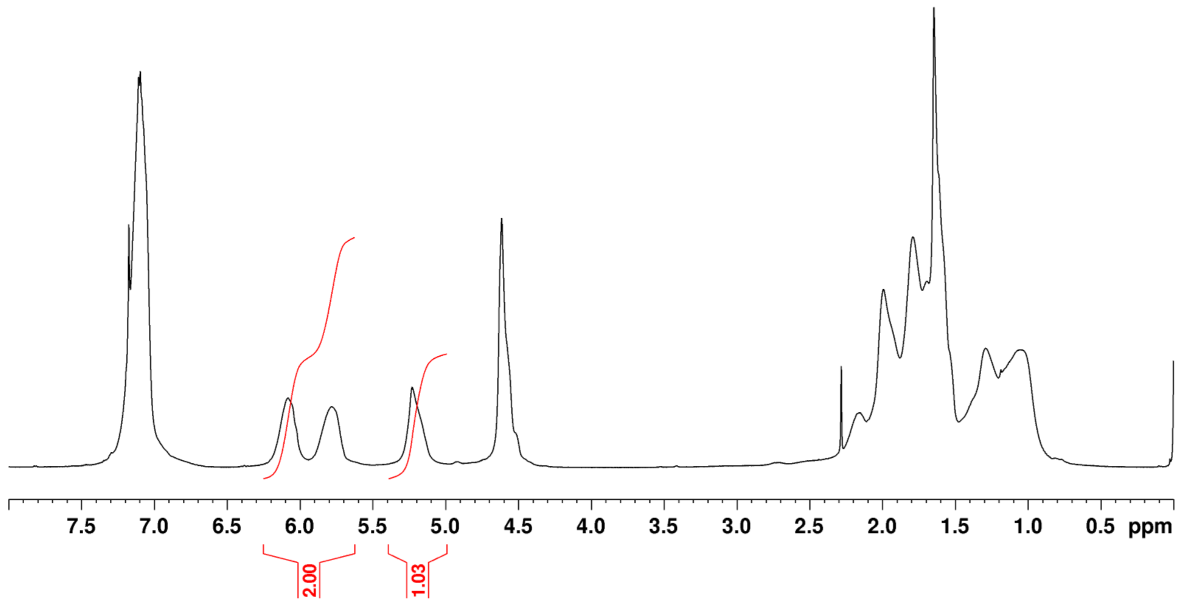

Figure S6. ^{1}H NMR (400 MHz, $CDCl_3$, 298 K) of PPBI copolymer from run **4**, Table 2.

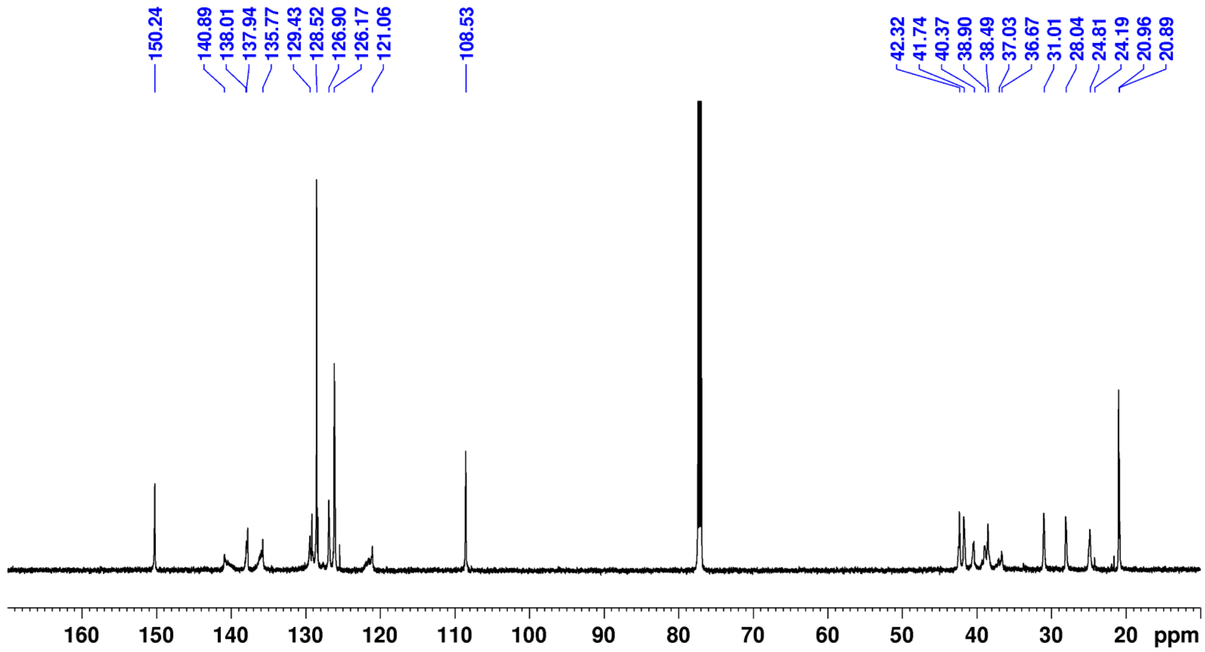

Figure S7. ^{13}C NMR (100 MHz, $CDCl_3$, 298 K) of PPBI copolymer from run **4**, Table 2.

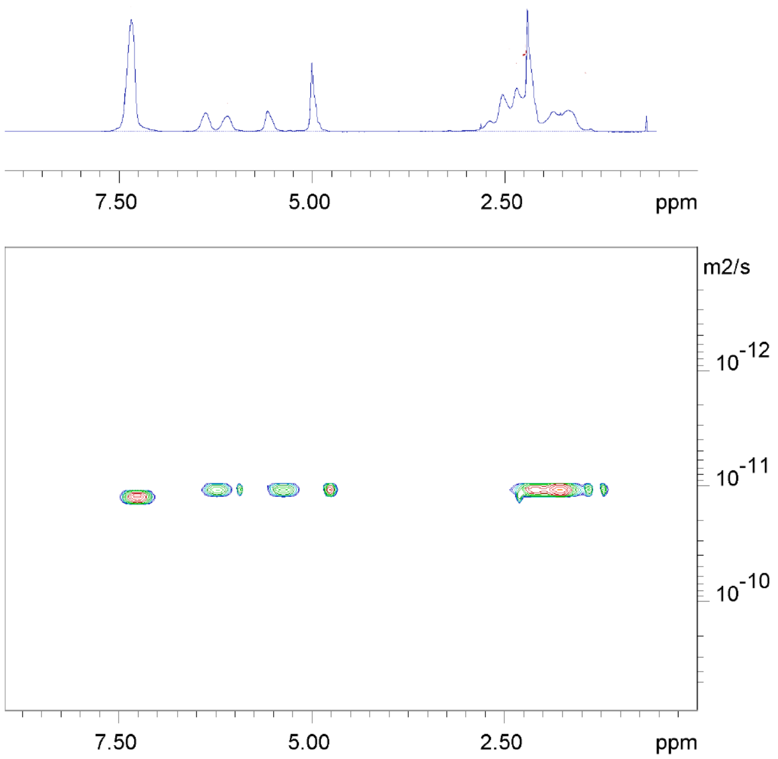

Figure S8. 2D DOSY NMR spectra (400 MHz, $CDCl_3$, 298 K) of PPBI copolymer from run **4**, Table 2.

3. Hydrogenation of poly(1PB)

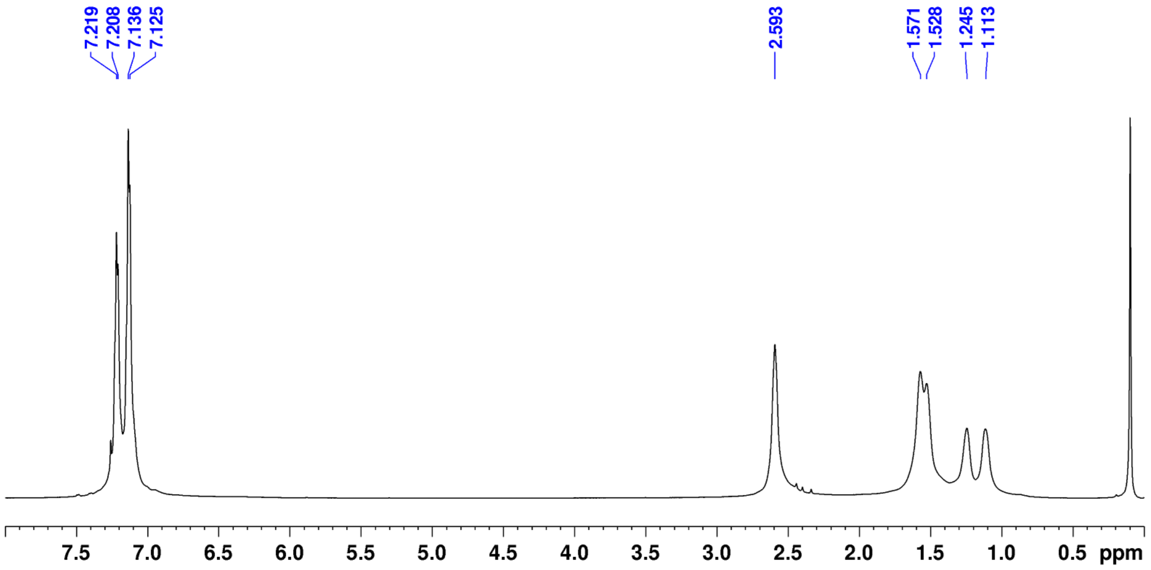

Figure S9. ^{1}H NMR (400 MHz, $CDCl_3$, 298 K) of 3,4-isotactic poly(1PB) after hydrogenation.

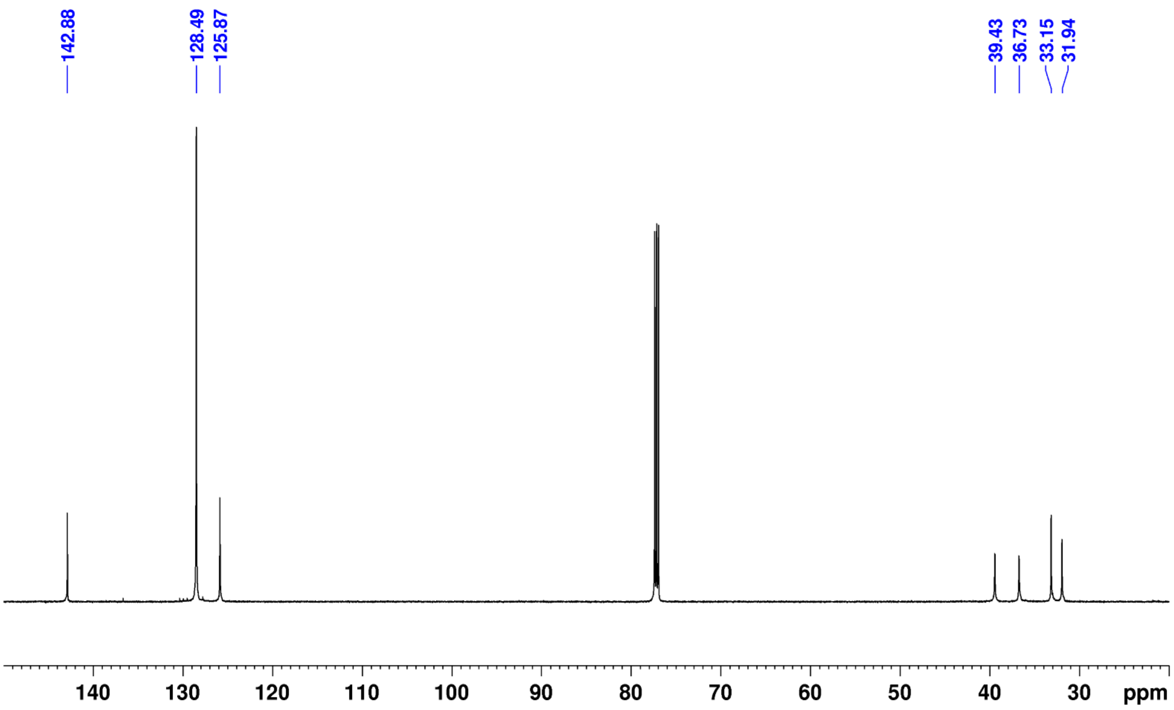

Figure S10. ^{13}C NMR (100 MHz, $CDCl_3$, 298 K) of 3,4-isotactic poly(1PB) after hydrogenation.

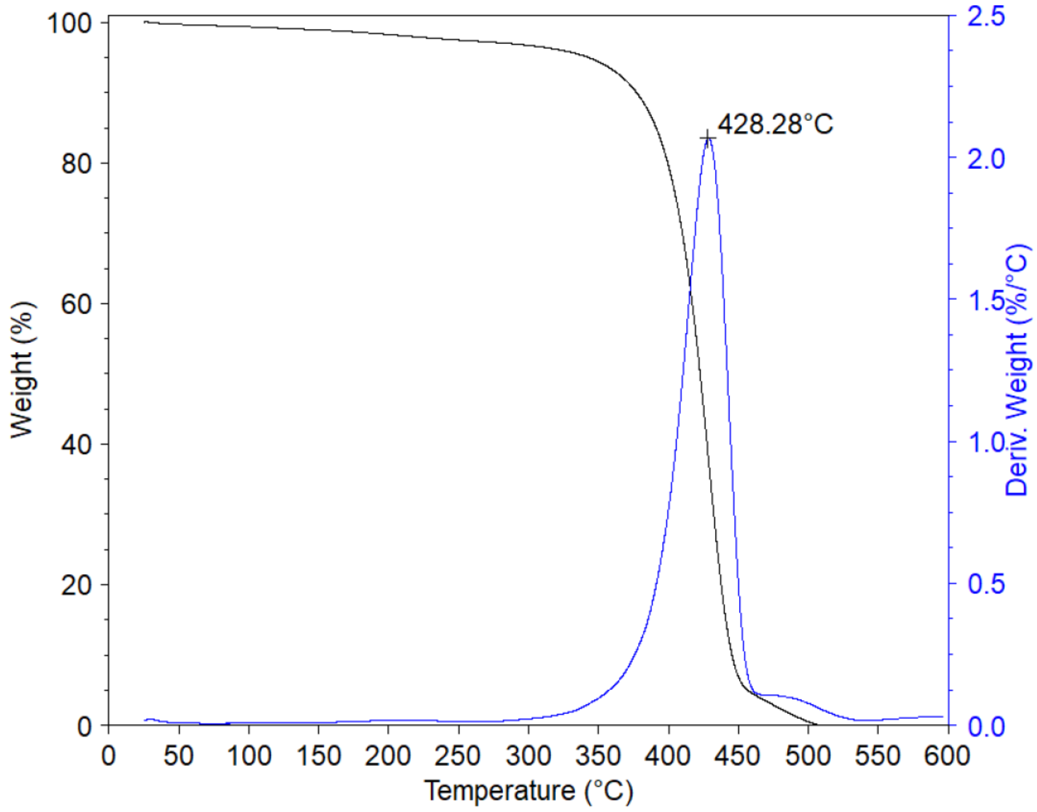

Figure S11. TGA traces of 3,4-isotactic poly(1PB) after hydrogenation.

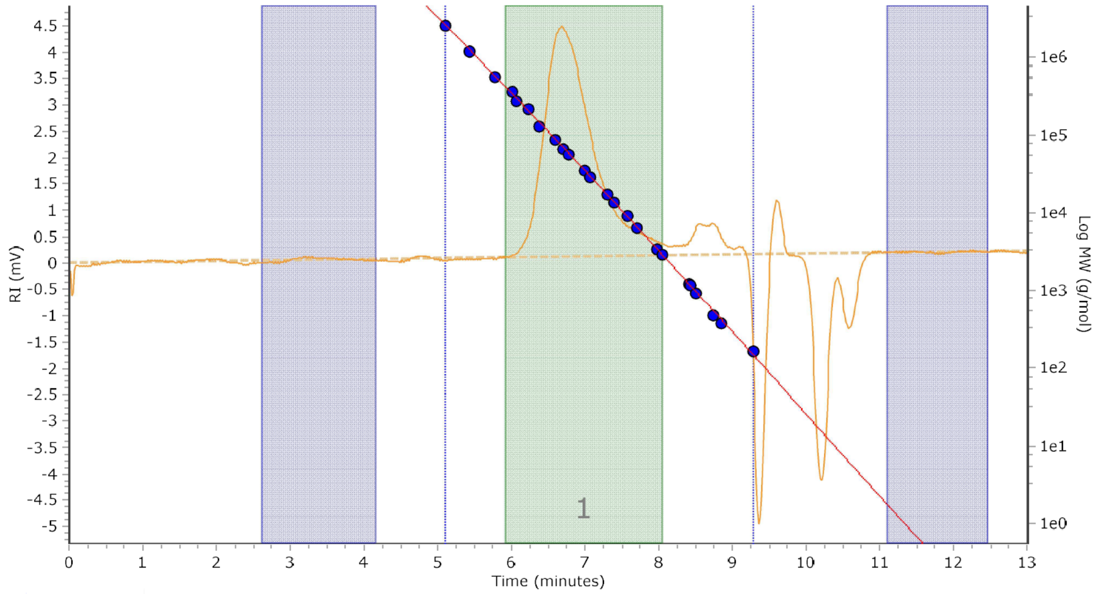

Peak	Mp (g/mol)	Mn (g/mol)	Mw (g/mol)	Mz (g/mol)	Mz+1 (g/mol)	Mv (g/mol)	PD
Peak 1	72573	30843	65556	98427	130318	60815	2.125

Figure S12. GPC curve of 3,4-isotactic poly(1PB) after hydrogenation.

4. DSC and XRD Analyses

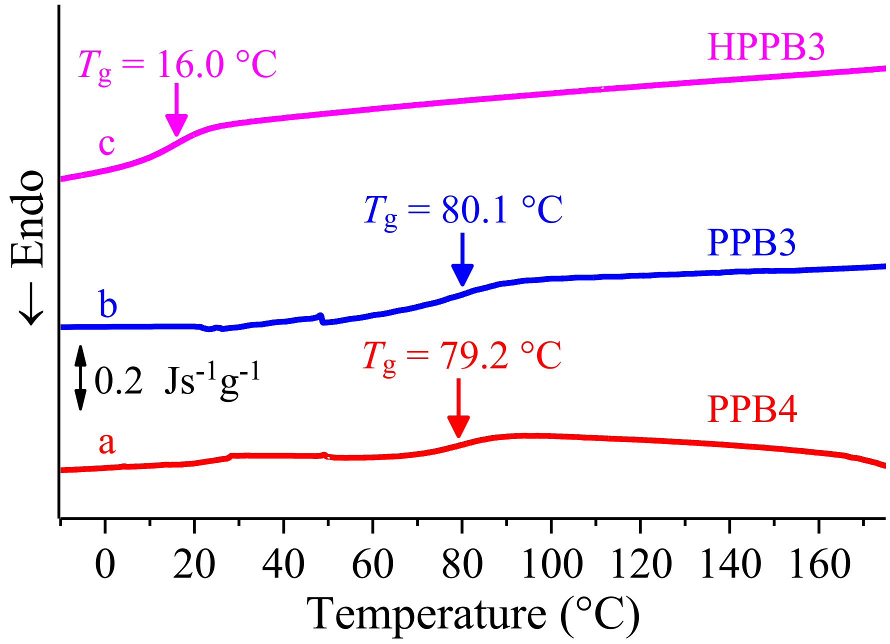

Figure S13. DSC curves recorded in the cooling step of as synthesized samples PPB4 (a), PPB3 (b) and of the sample PPB3 after hydrogenation (HPPB3, c).

Table S1. Thermal properties extracted from DSC analysis of PPB samples before (samples PPB3 and PPB4) and after hydrogenation (sample HPPB3).

**Sample**	T_{gI}; T_{gII}[a] (°C)	$T_{mI'}$; $T_{c'}$[b] (°C)	$\Delta H_{m'}$; $\Delta H_{c'}$[b] (J/g)	T_{mI}; T_{mII}[a] (°C)	ΔH_{mI}; ΔH_{mII}[a] (J/g)
PPB3	83.0; 83.7	-		127.1; 118	18.4; 2.2
PPB4	80.9; 80.4	-		123.2; 117	17.9; 2.0
**HPPB3**	34.4; 16.7	75.5; 109.1	2.6; 2.9	137.8;136.1	13.5; 2.1

[a]Glass transition (T_{gI}, T_{gII}), melting temperatures (T_{mI}, T_{mII}) and melting enthalpies (ΔH_{mI}, ΔH_{mII}) measured in the first (I) and second (II) DSC heating scans. [b]The hydrogenated sample shows in the first DSC heating scan a first endothermic peak at $T_{mI'} \approx 76$ °C ($\Delta H_{mI'} \approx 3$ J/g), followed by an exothermic peak at $T_{cI'} \approx 109$ °C ($\Delta H_{mI'} \approx 4$ J/g), due to melting of initial crystals and successive recrystallization into more stable crystals, melting at 136°C ($\Delta H_{mI'} \approx 6$g).

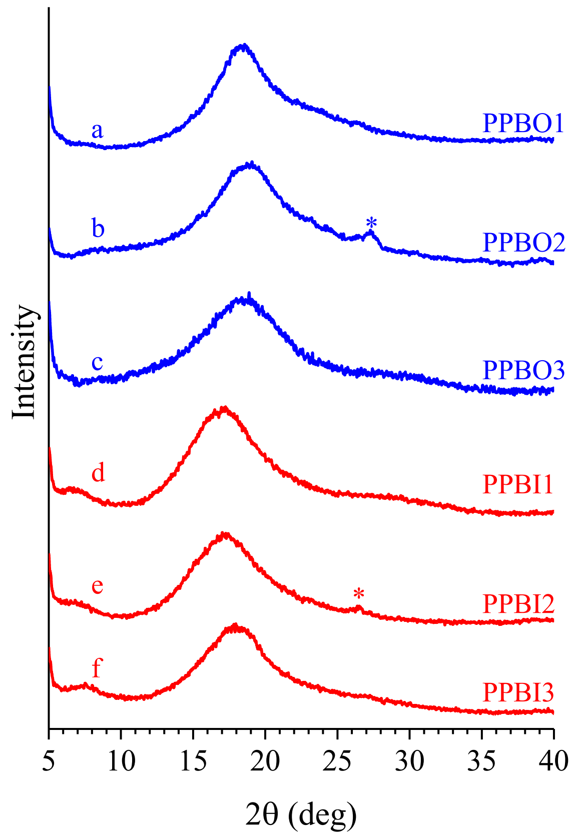

Figure S14. X-ray powder diffraction profiles of PPBO and PPBI samples. The stars at $2\theta \approx 27$ ° (curves b and e) indicate the presence of a small amount of some crystalline impurity (e.g. catalyst remnant).

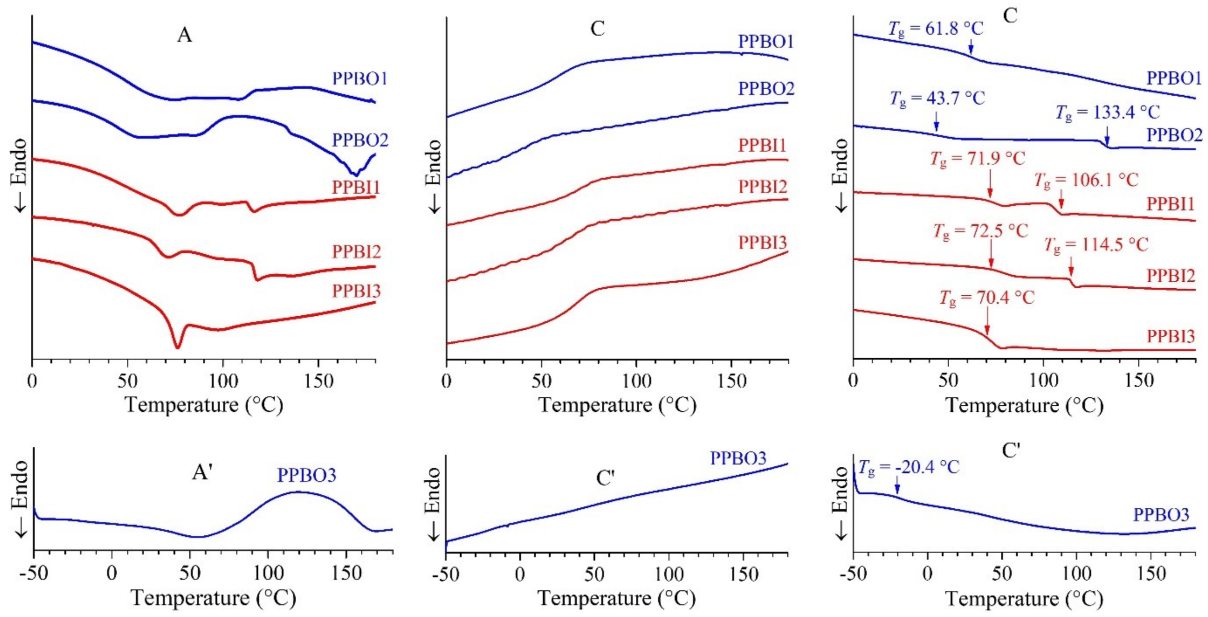

Figure S15. DSC curves recorded in the 1st heating, (A) successive cooling (B) and 2nd heating scans of as synthesized samples PPBO1, PPBO2, PPBI1, PPBI2, PPBI3 (A) and PPBO3 (A').

5. Thermogravimetric Analyses

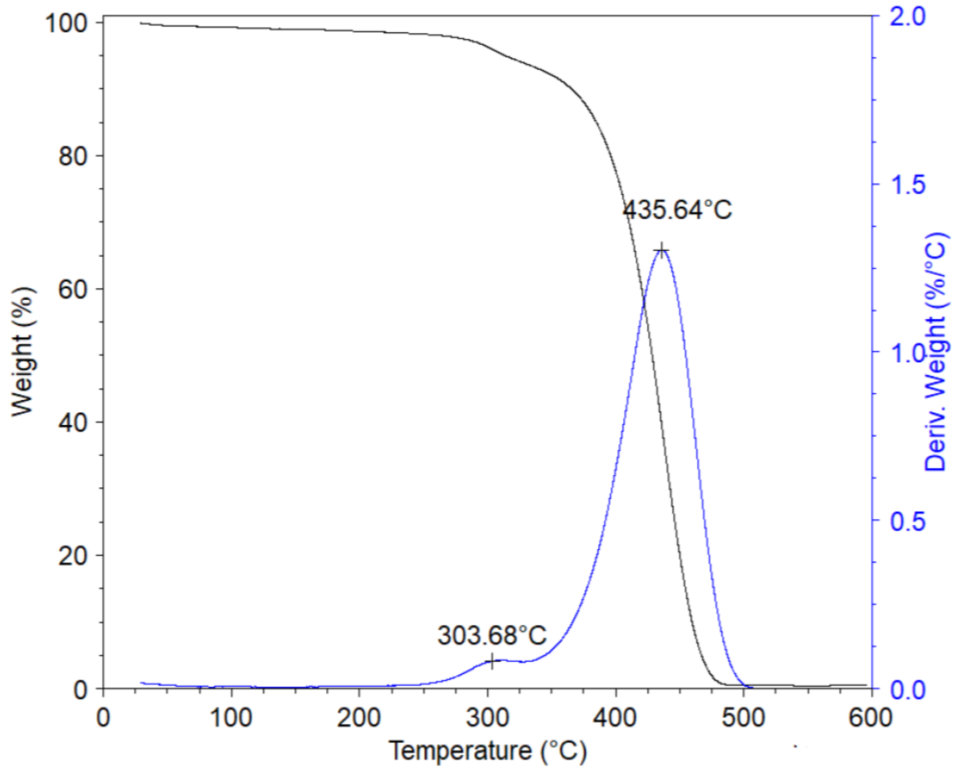

Figure S16. TGA traces of 3,4-isotactic poly(1PB) from run **3**, Table 1.

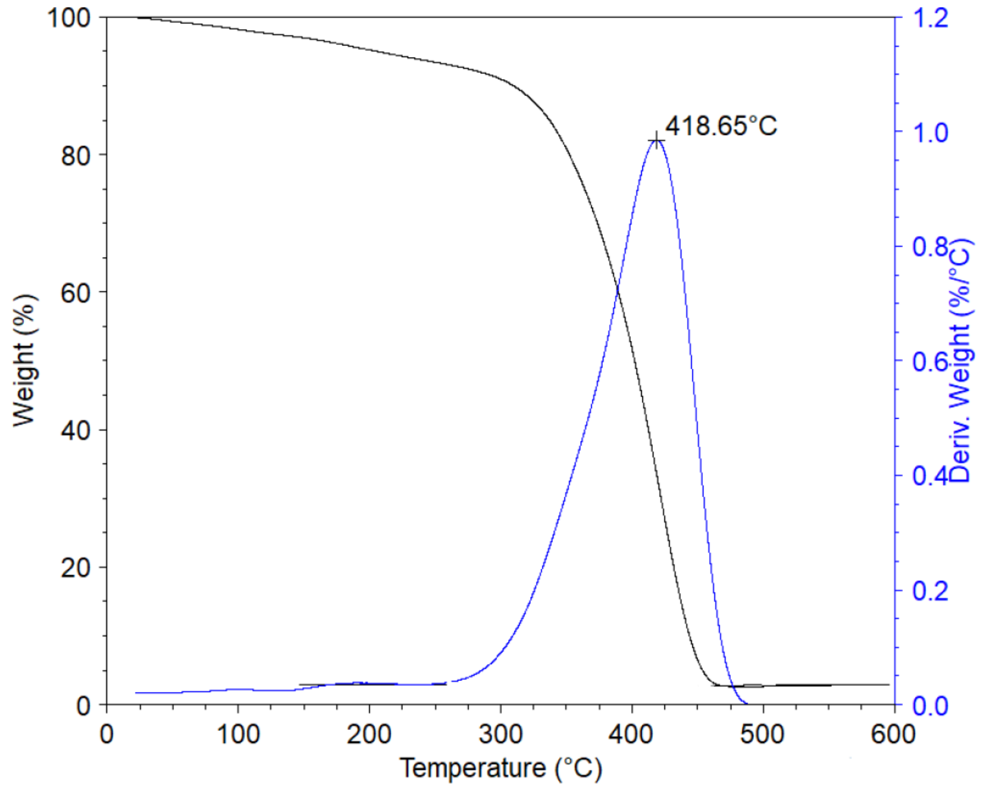

Figure S17. TGA traces of the PPBO copolymer from run **1**, Table 2.

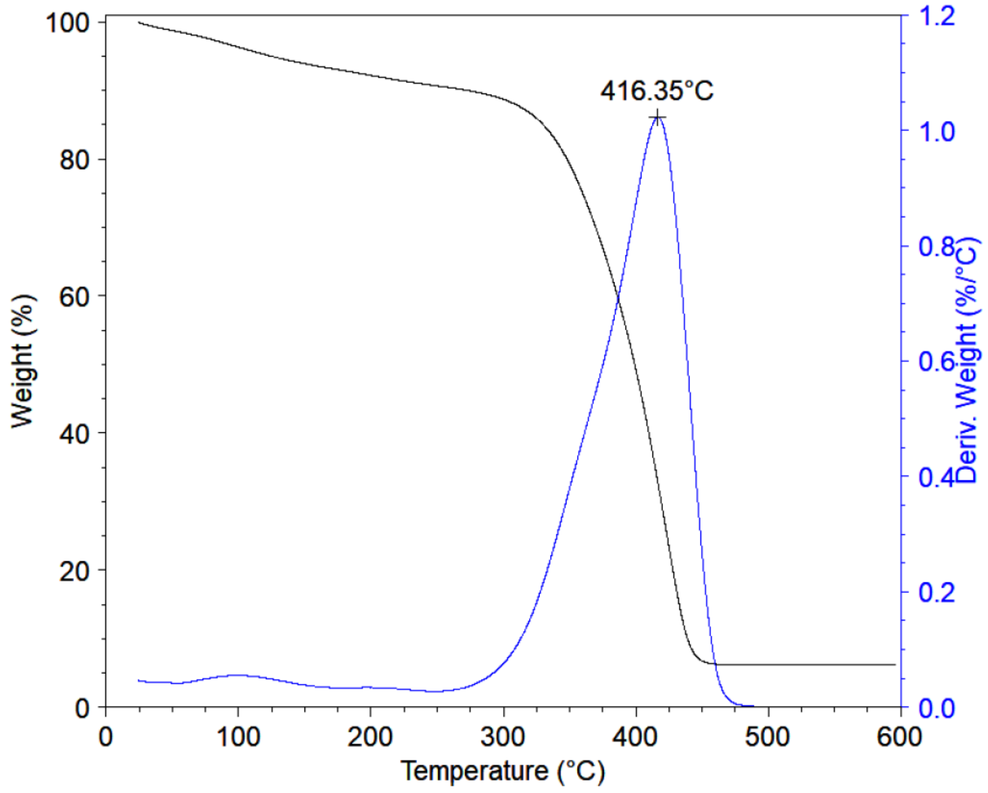

Figure S18. TGA traces of the PPBI copolymer from run **4**, Table 2.

6. GPC Analyses

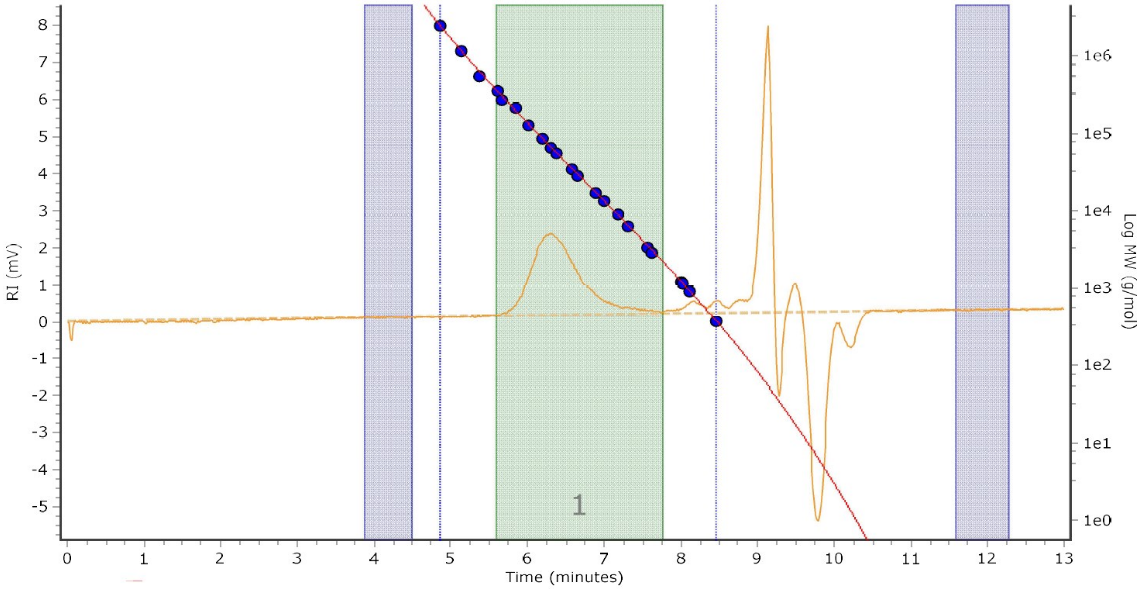

Peak	Mp (g/mol)	Mn (g/mol)	Mw (g/mol)	Mz (g/mol)	Mz+1 (g/mol)	Mv (g/mol)	PD
Peak 1	70580	30986	66260	100138	135260	61504	2,138

Figure S19. GPC curve of 3,4-isotactic poly(1PB) from run **3**, Table 1.

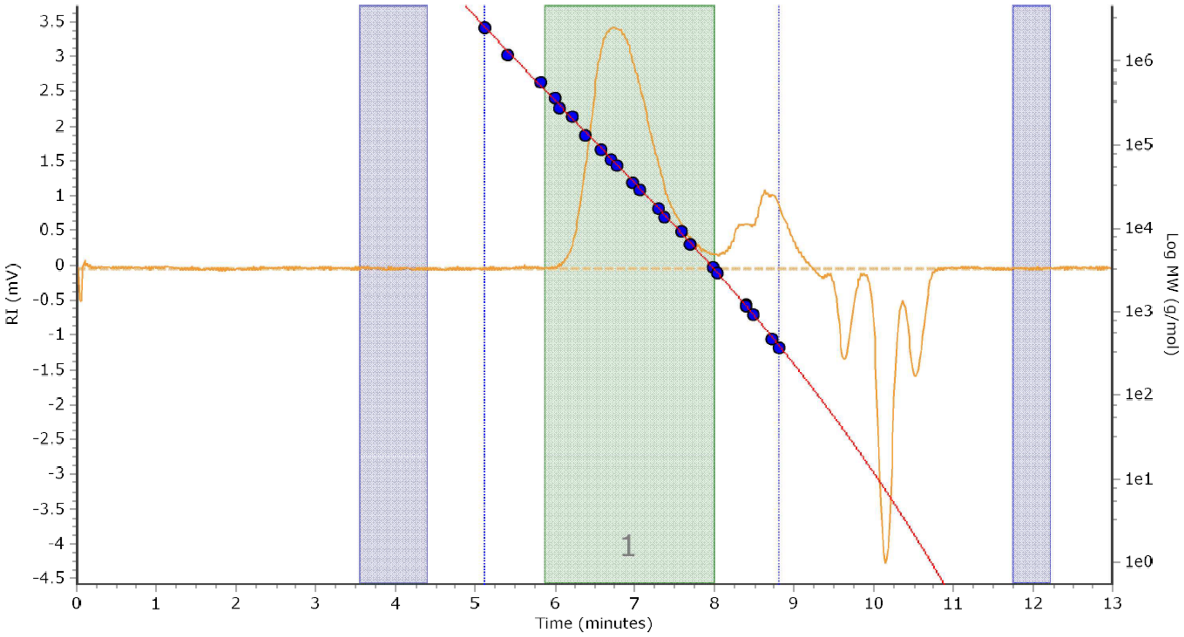

Peak	Mp (g/mol)	Mn (g/mol)	Mw (g/mol)	Mz (g/mol)	Mz+1 (g/mol)	Mv (g/mol)
Peak 1	65976	29551	59636	92682	125371	55072

PD
2.018

Figure S20. GPC curve of PPBO copolymer from run **2**, Table 2.

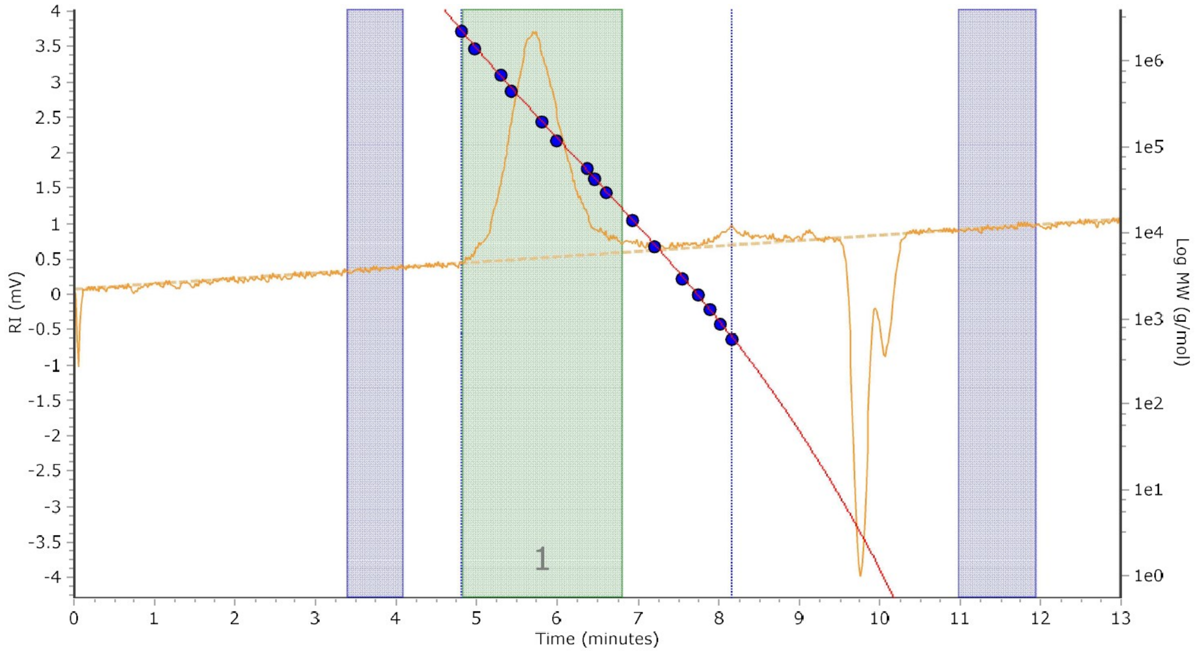

Figure S21. GPC curve of PPBI copolymer from run **4**, Table 2.

7. Mechanical properties

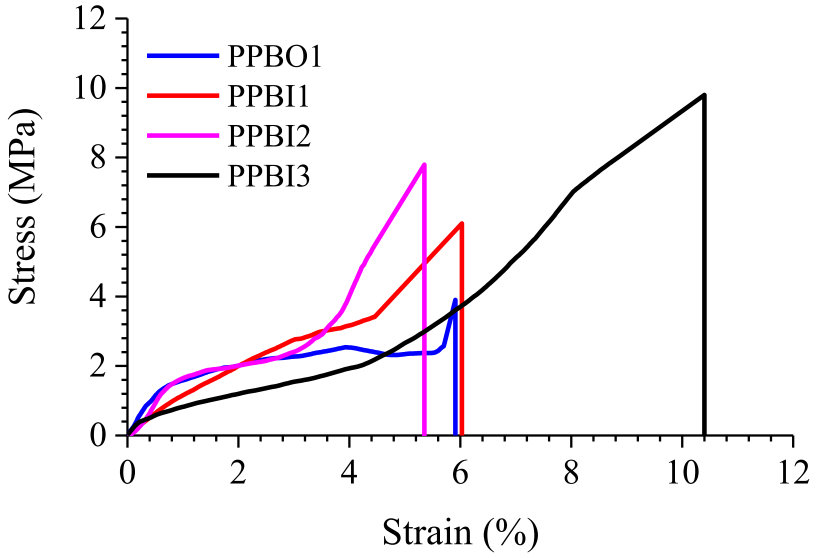

Figure S22. Stress-strain curves measured at room temperature using the deformation rate v/l_0 = 0.1 mm min^{-1} mm^{-1}, of the samples PPBO1, PPBI1, PPBI2 and PPBI3.

Table S2. Mechanical parameters extracted from stress-strain curves measured at 25 °C for the samples PPBO1, PPBI1, PPBI2 and PPBI3.

Samples	Terpene (O/IVC) content (mol%)	M_n (Kg/Mol)	*E* (MPa)	ε_y (%)	σ_y (MPa)	ε_b (%)	σ_b (MPa)
**PPBO1**	46 (O)	40.4	(37±9) 10	0.74±0.07	1.4±0.1	6±2	4±1
**PPBI1**	51 (IVC)	152.4	(27±1) 10	0.96±0.08	1.1±0.1	6±2	6±1
**PPBI2**	68 (IVC)	149.4	(3±1) 10^2	0.79±0.08	1.5±0.2	5±2	8±3
**PPBI3**	79 (IVC)	162.3	(13±8) 10	0.53±0.08	0.6±0.2	10±4	10±2

References

(1) Capacchione, C.; Manivannan, R.; Barone, M.; Beckerle, K.; Centore, R.; Oliva, L.; Proto, A.; Tuzi, A.; Spaniol, T. P.; Okuda, J. Isospecific Styrene Polymerization by Chiral Titanium Complexes That Contain a Tetradentate [OSSO]-Type Bis(Phenolato) Ligand. *Organometallics* **2005**, *24* (12), 2971–2982.

(2) Jiang, Y.; Kang, X.; Zhang, Z.; Li, S.; Cui, D. Syndioselective 3,4-Polymerization of 1-Phenyl-1,3-Butadiene by Rare-Earth Metal Catalysts. *ACS Catal.* **2020**, *10* (9), 5223–5229.
